# Overview of the Structure–Function Relationships of Mannose-Specific Lectins from Plants, Algae and Fungi

**DOI:** 10.3390/ijms20020254

**Published:** 2019-01-10

**Authors:** Annick Barre, Yves Bourne, Els J. M. Van Damme, Pierre Rougé

**Affiliations:** 1 UMR 152 PharmaDev, Institut de Recherche et Développement, Faculté de Pharmacie, Université Paul Sabatier, 35 Chemin des Maraîchers, 31062 Toulouse, France; annick.barre@univ-tlse3.fr; 2 Centre National de la Recherche Scientifique, Aix-Marseille Univ, Architecture et Fonction des Macromolécules Biologiques, 163 Avenue de Luminy, 13288 Marseille, France; yves.bourne@afmb.univ-mrs.fr; 3 Department of Biotechnology, Faculty of Bioscience Engineering, Ghent University, Coupure links 653, B-9000 Ghent, Belgium; ElsJM.VanDamme@UGent.be

**Keywords:** lectin, plant, fungi, mannose-binding specificity, structure, function, use as tools

## Abstract

To date, a number of mannose-binding lectins have been isolated and characterized from plants and fungi. These proteins are composed of different structural scaffold structures which harbor a single or multiple carbohydrate-binding sites involved in the specific recognition of mannose-containing glycans. Generally, the mannose-binding site consists of a small, central, carbohydrate-binding pocket responsible for the “broad sugar-binding specificity” toward a single mannose molecule, surrounded by a more extended binding area responsible for the specific recognition of larger mannose-containing *N*-glycan chains. Accordingly, the mannose-binding specificity of the so-called mannose-binding lectins towards complex mannose-containing *N*-glycans depends largely on the topography of their mannose-binding site(s). This structure–function relationship introduces a high degree of specificity in the apparently homogeneous group of mannose-binding lectins, with respect to the specific recognition of high-mannose and complex *N*-glycans. Because of the high specificity towards mannose these lectins are valuable tools for deciphering and characterizing the complex mannose-containing glycans that decorate both normal and transformed cells, e.g., the altered high-mannose *N*-glycans that often occur at the surface of various cancer cells.

## 1. Introduction

Protein-carbohydrate interactions are part of the most efficient signaling pathways occurring inside living organisms or between living organisms and their environment. Lectins or Carbohydrate-Binding Agents (CBAs) are proteins that have specialized in the specific recognition of carbohydrates during the evolution of all living organisms. The large family of carbohydrate-binding proteins contains a large variety of carbohydrate-binding domains (CBDs), each with one or more carbohydrate-binding sites (CBSs) which specifically recognize simple or more complex sugars. Depending on the lectin, the carbohydrate-binding domains belong to distinct structural scaffolds usually organized in homo- or hetero-dimeric or tetrameric structures [1]. According to the nature and the organization of their domains, plant and fungal lectins have been classified in two groups of lectins, (1) lectins exclusively composed of carbohydrate-binding domains and (2) chimerolectins composed of a carbohydrate-binding domain linked to another domain(s) devoid of any carbohydrate-binding properties [1]. With respect to their binding properties, plant and fungal lectins can be subdivided in different groups, such as for example Man-specific lectins, Gal/GalNAc-specific lectins, and Fuc-specific lectins [2]. However, the binding of lectins towards simple sugars is probably not really relevant. It is more realistic to assume that lectins will interact with the more complex *N*-glycan chains that decorate the cell surface of all living organisms [3]. In addition, the idea has progressively emerged that, besides lectins which are abundantly distributed in storage tissues like seeds and bulbs and play a defensive/protective role, other less abundant lectins participate in more discrete carbohydrate recognition processes equally necessary for the proper functioning of the living organisms [4]. In this respect, the discovery of Nictaba, a lectin localized in the nucleus of tobacco (*Nicotiana tabacum*) cells, represents a milestone in our vision of the function devoted to plant and fungal lectins in vivo [5].

Owing to the huge amount of structural and functional data that have been accumulated for several decades these carbohydrate-binding proteins from plants and fungi have become a tool to decipher the structure–function relationships inherently associated to protein macromolecules. In this respect, lectins involved in the specific recognition of mannosyl residues, the so-called mannose-binding lectins, represent an important group of functional proteins taking into account the widespread distribution of mannose-containing *N*-glycans of the *N*-acetyllactosamine type and high-mannose type. The present review aims to present an exhaustive overview that summarizes all published informations related to the structure–function relationships of mannose-specific lectins from plants and fungi, and their possible applications as analytical and therapeutic tools for biomedical research.

## 2. Diversity of Mannose-Binding Lectins in Higher Plants

To date, lectins with a mannosyl-binding specificity have been identified in many different plant families, including monocotyledonous as well as dicotyledonous species (Table 1). Among the monocot families, research has focused on the Liliaceae and Amaryllidaceae [6], whereas the Fabaceae family occupies a predominant position in the dicot group [6]. Following to the pioneering work of Agrawal & Goldstein [7], who reported that concanavalin A (Con A), the lectin from Jack bean (*Canavalia ensiformis*) seeds, was easily retained by simple filtration through a column containing cross-linked dextran gel (Sephadex, Pharmacia) and subsequent desorbtion by the addition of glucose or mannose to the eluting buffer, both Con A and many other mannose-specific lectins (Table 1) were easily purified using a single affinity chromatography step. Mannose-specific lectins were also successfully isolated from different algae, mushrooms and lower plant species [8]. Moreover, some mannose-specific lectins from red algae specifically recognize the core (α1-6)-fucosylated *N*-glycans of cancer cells and can be used as biomarkers for the detection of cancer glycoforms [9]. In this respect, they resemble LcA from *Lens culinaris*, PsA from *Pisum sativum* and LoL-I from *Lathyrus ochrus*, which show strong binding to core-fucosylated mono- and bi-antennary *N*-glycans [10,11].

## 3. Structural Organization of the Plant, Algal and Fungal Mannose-Binding Lectins

### 3.1. Structure of Mannose-Specific Plant Lectins

Mannose-specific lectins from plants essentially belong to three distinct structural scaffolds that assemble in different ways to generate more complex oligomeric structures:

#### 3.1.1. The β-Sandwich Fold

**Figure 1:**
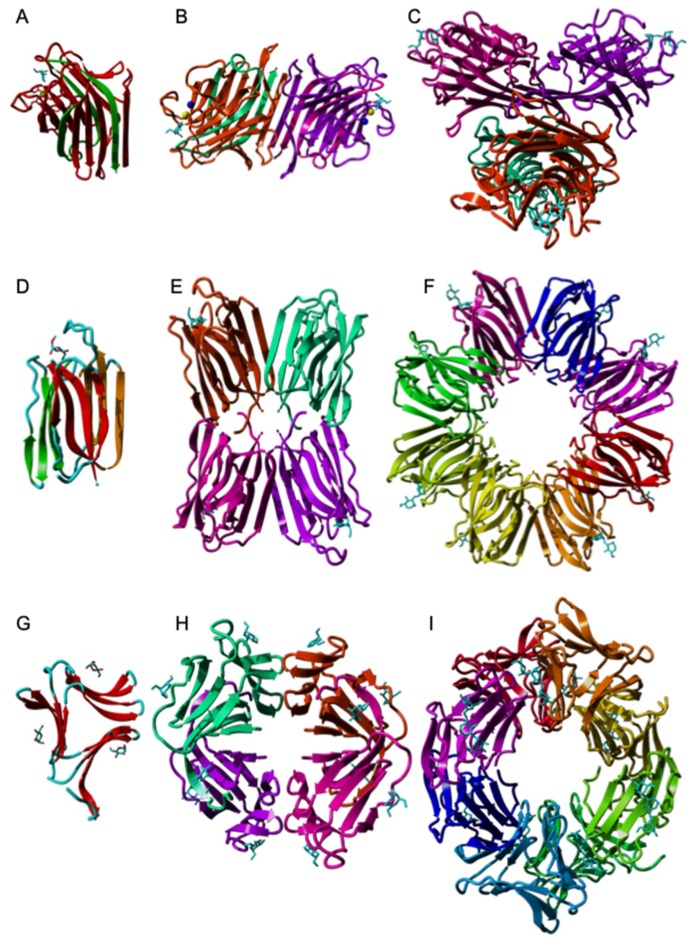
Structural diversity of the mannose-binding lectins. (**A**). Two-chain lectin protomer of *Lathyrus ochrus* (PDB code 1LOE [48]). Light chain and heavy chains are colored green and red, respectively. (**B**). Homodimeric organization of the *L. ochrus* isolectin-I (1LOE). The light and heavy chains of the dimer are colored differently. (**C**). Homotetrameric organization of Con A (PDB code 3CNA). The four single-chain protomers are shown in different colors. (**D**). The β-prism organization of the artocarpin protomer from *Artocarpus integrifolia* (PDB code 1J4S). The three bundles of β-strands forming the β-prism are colored green, red and orange, respectively. (**E**). Homotetrameric organization of artocarpin from *A. integrifolia* (1J4U). The β-prism protomers are colored differently. (**F**). Homooctameric organization of Heltuba from *Helianthus tuberosus* (1C3M) [81]. The β-prism protomers are colored differently. (**G**). The β-prism II organization of the protomer of GNA from *Galanthus nivalis* (PDB code 1MSA). (**H**). Organization of the β-prism II protomers in the GNA tetramer (PDB code 1MSA). (**I**). Hexameric structure of the tarin lectin from *Colocasia esculenta* (PDB code 5T20). The six β-prism-folded protomers are colored differently.

The jelly roll scaffold occurring in legume lectins (Fabaceae) consists of either a single or two polypeptide chains. In two-chain lectins, the light (α) and heavy (β) chains made of six and seven strands of antiparallel β-sheet, respectively, non-covalently associate in a β-sandwich protomer (Figure 1A). Protomers associate by non covalent bonds to give the homodimeric lectins of the Vicieae tribe, e.g., pea lectin (*Pisum sativum* agglutinin PsA) [57], lentil lectin (*Lens culinaris* agglutinin LcA) [156], yellow vetch lectin (*Lathyrus ochrus* lectin Lol) [48] (Figure 1B), and the faba bean lectin (*Vicia faba* agglutinin VfA or favin) [63] (Figure 1B). In contrast, the Man-specific lectin from *Lathyrus sphaericus* consists of an uncleaved single chain protomer [51]. The single-chain protomers associate into homotetramers. Examples are the mannose-binding lectins characterized in the tribes Baphieae (*Bowringia mildbraedii* agglutinin BMA) [17], Dalbergieae (*Centrolobium tomentosum* lectin CTL [29], *Pterocarpus angolensis* lectin PAL [58]), Diocleae (Con A [23,157] (Figure 1C), *Cymbosema roseum* CRL [33], *Dioclea grandiflora* lectin Con GF [25], and other *Dioclea* sp. lectins). Dimeric lectins such as PsA, possess two identical mannose-binding sites whereas tetrameric lectins like Con A, exhibit four mannose-binding sites. Gal/GalNAc-specific lectins from other legume tribes such as the soybean agglutinin SBA (*Glycine max*) from the Glycinae tribe (PDB code 1SBF) [158], the peanut agglutinin PNA (*Arachis hypogaea*) from the Aeschynomeneae (PDB code 2PEL) [159], the coral tree lectin EcorL (*Erythrina corallodendron*) from the Erythrinae tribe (PDB code 1AXY) [160], and the kidney bean leucoagglutinin PHA-L (PDB code 1FAT) [161] and erythroagglutinin PHA-E (PDB code 3WCR) [162], (*Phaseolus vulgaris*) belonging to the Phaseolae tribe, all strikingly resemble Con A and other Diocleae lectins but differ in the topological organization for the single-chain protomers that constitute the lectin.

#### 3.1.2. The β-Prism I Fold

The β-prism I scaffold serves as a building block for the mannose-binding lectins in seeds of the Moraceae such as artocarpin, the lectin from the Jackfruit (*Artocarpus* integrifolia) seeds which serves as a prototype for this group [163]. The β-prism I scaffold consists of three bundles of four antiparallel β-strands forming three Greek keys 1, 2 and 3, arranged into a β-prism structure along a longitudinal axis (Figure 1D). Depending on the lectins, a posttranslational proteolytic cleavage between the β-strands β1 and β2 of Greek key 1 occurs during seed ripening, to liberate the light α-chain with a terminal Gly1 residue exhibiting a free H_2_N- group, and the heavy β-chain comprising the rest of the β-prism structure. This proteolytic cleavage occurs in the Gal/GalNAc-specific homotetrameric lectins of Moraceae, such as jacalin (Figure 1E) (PDB code 1JAC) [164], the MPA lectin from Osage orange (*Maclura pomifera*) seeds (PDB code 1JOT) [165], and the Gal/GalNAc-specific lectin Morniga-G from the bark of blackberry (*Morus nigra*) [80]. However, the Man-specific lectins from the Moraceae family, e.g., artocarpin from Jackfruit [163] and Morniga-M from blackberry [166], consist of an uncleaved single-chain β-prism polypeptide chain. Similarly, Heltuba, the lectin from the Jerusalem artichocke (*Helianthus tuberosus*), also consists of a single-chain β-prism polypeptide chain made of 8 β-prisms non-covalentlty associated around a central axis to form a flattened star-shaped architecture comprising 8 identical carbohydrate-binding sites (Figure 1F) [81].

#### 3.1.3. The β-Prism II Fold

The β-prism II scaffold was first identified in GNA, the mannose-specific lectin isolated from the bulbs of snowdrop (*Galanthus nivalis*), a plant species belonging to the monocot family Amaryllidaceae [110]. The scaffold consists of three bundles of four β-strands arranged into a flattened β-prism structure around a central pseudoaxis (Figure 1G). A carbohydrate-binding site occurs in a groove located at the center of the bundle of β-strands forming each β-sheet. The monocot-specific lectins result from the non-covalent association of four β-prism II scaffolds. Depending on the lectin, four identical β-prism II of 12 kDa form a homotetramer, e.g., in GNA (Figure 1H) [167], whereas other lectins consist of heterotretramers built up from the symmetrical association of two 12 kDa and two 14 kDa β-prism subunits, e.g., the Araceae lectins [6]. Usually, all three carbohydrate-binding sites occurring in each β-prism scaffold are readily functional but in a few lectins, one or two carbohydrate-binding sites are apparently inactive due to point mutation(s) in key residues involved in the H-bonding of mannose. Tarin from *Colocasia esculenta* assembles into homohexameric structures made of 6 β-prism scaffolds [168] (Figure 1I).

**Figure 2:**
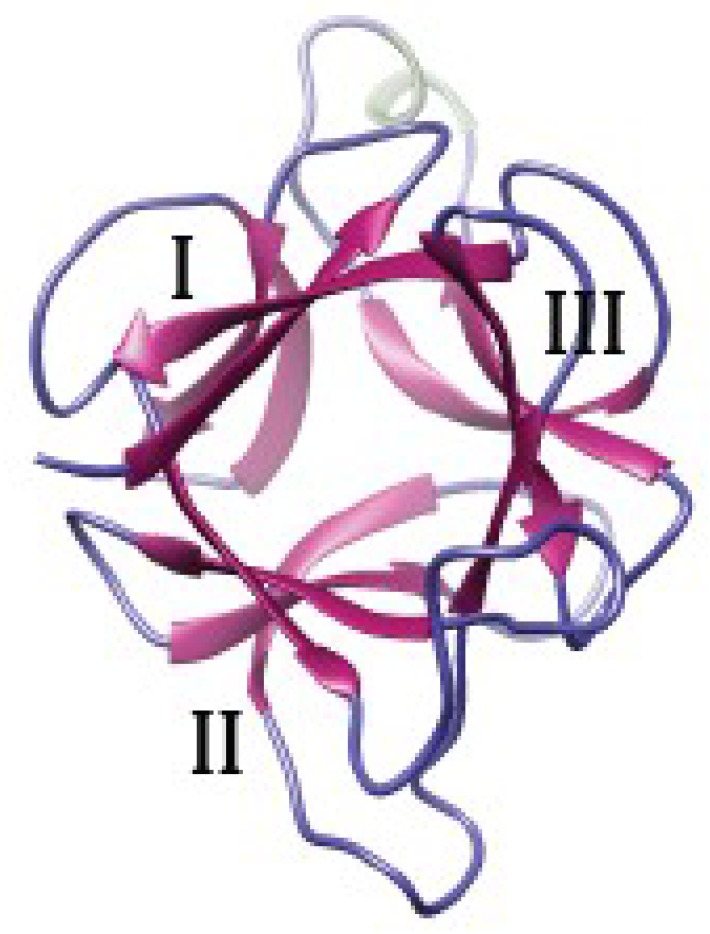
Three-dimensional models for the EUL domain of EUL-domains of rice lectin Orysata, showing the β-trefoill organization made of three bundles of antiparallel β-sheets (I, II, III).

The β-trefoil scaffold, another β-prism II scaffold, has been primarily identified in type II Ribosome-Inactivating Proteins (RIP-II), in amaranthin, a T antigen-specific lectin from amaranth (*Amaranthus caudatus*) [169], and it also occurs in the stress inducible lectins composed of EUL (*Euonymus* lectin) domains, such as the lectins from rice (*Oryza sativa*) and *Arabidopsis* [170]. The β-trefoil scaffold consists of six β-hairpins arranged around an approximate three-fold symmetry axis, linked to extended loops that simulate the three lobes of a trefoil leaf (Figure 2). The Man-binding sites are located in the shallow depressions of the β-strands but, usually not all binding sites are functional. 

An unexpected four-bladed β-propeller structure was found to occur in a PA_2_ albumin from chickpea (*Cicer arietinum*), which displays a well documented hemagglutinating activity most probably related to a lectin with an unusual hemopexin fold [171].

### 3.2. Structure of Mannose-Specific Algal Lectins

**Figure 3:**
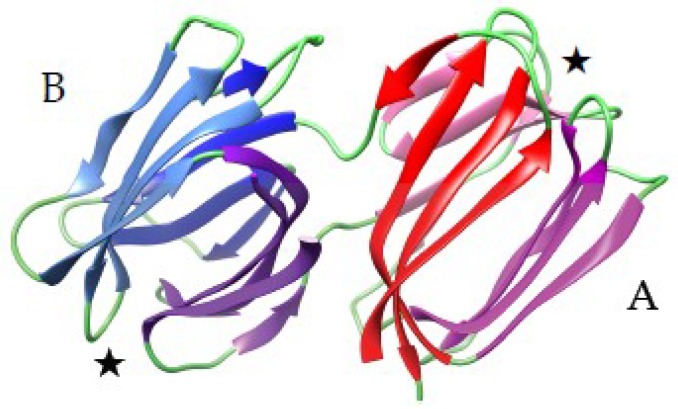
Three-dimensional model of griffithsin (PDB code 2GTY), showing the β-prism organization made of three four-stranded β-sheets in each monomer. The four stranded β-sheets are colored red, pink and magenta in monomer (**A**), and blue, light blue and purple in monomer (**B**), respectively. The stars indicate the localization of the carbohydrate-binding sites in each monomer.

The mannose-specific lectin griffthsin from the red alga *Griffthsia* sp., consists of a domain- swapped dimer made of two protomer exhibiting the β-prism I fold, that closely resembles to the jacalin-related lectin organization (PDB code 2GTY) [140]. Swapping results from the participation of two β-strands of one molecule in the completion of the three four-stranded sheets forming the β-prism of the other molecule, and vice versa. As a result of this swapping, both molecules in the dimer consist of a complete β-prism organization (Figure 3).

In spite of a high number of cloned and sequenced lectins from different species of red and green algae, their three-dimensional organization(s) were poorly investigated and still remain unknown. Their amino acid sequences readily differ from that of griffithsin and, most probably, they also differ from griffithsin by their three-dimensional structure and monomer organization. 

### 3.3. Structure of Mannose-Specific Fungal Lectins

**Figure 4:**
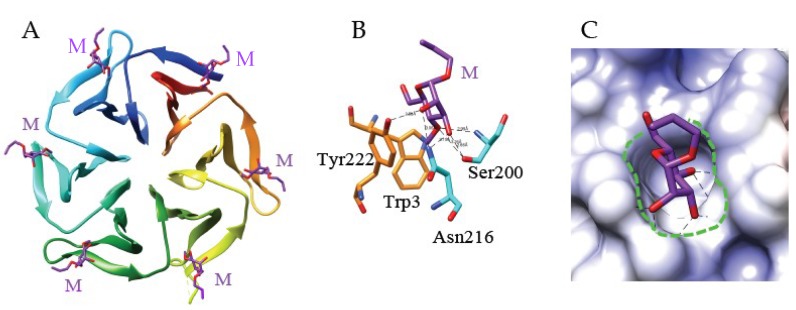
(**A**). Beta-propeller organization of tectonin 2 from the mushroom *Laccaria bicolor* in complex with allyl-α4-methyl-mannoside. The lectin consists of 6 antiparallel strands of β-sheet (colored differently) organized in 6 blades around the axis of the β-propeller. The allyl-mannoside residues (M) anchored to the carbohydrate-binding sites of the lectin are colored purple (PDB code 5FSC) (**B**). Sixth mannose-binding site of tectonin 2 in complex with allyl-α4-methyl-mannoside. Hydrogen bonds connecting the monosaccharides to the amino acid residues Ser200, Asn216 and Tyr222, forming the monosaccharide-binding site are represented by black dashed lines. Aromatic residues Trp3 and Tyr222, paticipating in stacking interactions with the sugar ring are colored orange. The molecular surface of the lectins is colored dark grey and their extended oligosaccharide-binding areas are delineated by white dashed lines. (**C**). The shallow depression corresponding to the monosaccharide-binding site that accommodates the allyl-mannoside residue (colored purple) at the molecular surface (colored according to the oulombic charges) of tectonin 2, is delineated by a green dashed line.

Mannose-specific lectins isolated from fungi result from the non-covalent association of different structural scaffolds resulting in more complex oligomeric structures:

An unusual six-bladed β-propeller organization built up from 4-stranded anti-parallel β-sheets was identified in tectonin 2, a lectin from the mushroom *Laccaria bicolor* AAL (PDB code 5FSC), that specifically recognizes O-methylated glycans [148] (Figure 4).

A similar 6-bladed β-propeller structure was observed in the fucose-binding lectins from the bacteria *Ralstonia solanacearum* [172], *Photorhabdus luminescens* [173], *Photorhabdus asymbiotica* [174], as well as in the tachylectin from the Japanese horseshoe crab *Tachypleus tridentatus* [175]. However, the β-propeller scaffold is not specific for the fucose-binding property since a β-propeller structure was shown to occur in other lectins with quite different sugar-binding specificities, e.g., the Neu5Ac- and GlcNAc-specific lectins from the mushrooms *Psathyrella velutina* [176] and *Psathyrella asperospora* [177], and the lectin Bambl from the bacterium *Burkholderia ambifaria*, which specifically interacts with the lewis x antigen, the blood H type 1 and H type 2 tetrasaccharides and the blood group B epitope [178].

**Figure 5:**
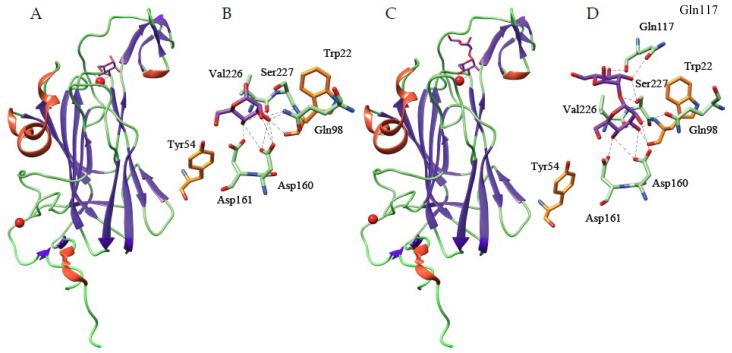
(**A**,**C**). Beta-sandwich organization of Flo5 from the yeast *Saccharomyces cerevisiae* in complex with mannose (**A**) (PDB code 2XJP) and α1,2-mannobiose (**C**) (PDB code 2XJS). The mannose-binding N-terminal domain of Flo5 consists of two strands of β-sheet forming a β-sandwich structure. (**B**). Network of hydrogen bonds anchoring mannose (colored purple) to the amino acid residues forming the carbohydrate-binding site located at the top of the β-sandwich. Two stacking interactions of the pyranose ring of mannose with aromatic residues Tyr54 and Trp228 (colored orange), complete the interaction. (**D)**. Network of hydrogen bonds anchoring α1,2-mannobiose (colored purple) Flo5, showing additional hydrogen bonds anchoring α1,2-mannobiose to Gln117 and Ser 227 residues. Residues Asp160, Asp161, Val226 and Trp228, also serve as ligands for a Ca^2+^ ion (colored red in **A** and **C**) located at the bottom of the mannose-binding pocket.

The β-sandwich scaffold is another structural scaffold found in the mannose-binding *N*-terminal domain of flocculins Flo1 and Flo5 from *Saccharomyces cerevisiae*, and Flo1 from *S. pasteurianus* [150,151]. These surface-adhesins possess a *N*-terminal domain that readily accomodates Man and α1,2-mannobiose via a network of hydrogen bonds and stacking interactions with aromatic residues, very similar to those occurring in Man-specific lectins of higher plants (Figure 5). Most of the aminoacid residues involved in the binding of mannose also serve as ligands for a Ca^2+^ ion located at the bottom of the mannose-binding site. The mannose-binding activity of Flo1 and Flo5 proteins plays a key role in the self-recognition processes occurring during the growth of the yeasts.

**Figure 6:**
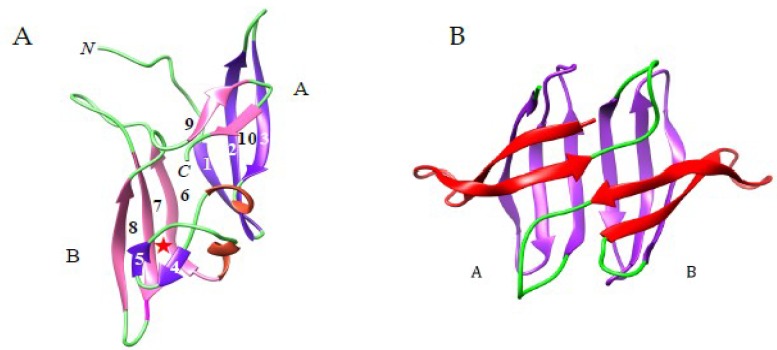
(**A**). Beta-sandwich organization of NcCVNH from *Neurospora crassa* (PDB code 2JZL), showing the two-swapped domains A (colored purple) and B (colored pink). Strands of β-sheet are numbered 1–10. *N* and *C* indicate the *N*-terminal and C-terminal extremities of the polypeptide chain, respectively. The mannose-binding site has been identified at the top of domain B (red star ★). (**B**). Ribbon diagram showing the structural organization of the two-domain (A and B) cyanobacterial microvirin from *Microcystis aeruginosa* (PDB code 2YHH) The β-strands, β-hairpins and turns, are colored purple, red and green, respectively.

The cyanovirin-fold (CVN-fold) also occurs as a structural scaffold identified in the cyanovirin-N family of mannose-binding fungal lectins, including the ascomycetous fungi *Ceratopteris richardii* (CrCVNH), *Neurospora crassa* (NcCVNH) and *Tuber borchii* (TbCVNH) [155]. The NcCVNH lectin consists of a two swapped domains polypeptide chain of 111 amino acids, built up from a domain A of 56 residues (residues 1–42 and residues 100–111), and a domain B of 57 residues (residues 43–99). According to the swapping occurring between both domains, domain A comprises the triple-stranded β-sheet (β1, β2, β3) associated to the β-hairpin (β9, β10), whereas domain B consists of the triple-stranded sheet (β6, β7, β8) associated to the β-hairpin (β4, β5) (Figure 6A). Other CrVNH and TbCVNH exhibit a very similar organization. 

It is notheworthy that most of the Man-specific lectins identified in bacteria consist of the so-called CVN family fold (Table 2), which comprises cyanovirin, actinohivin, and microvirin occurring in cyanobacteria (ex blue-green algae) as a two swapped domains polypeptide chain, each domain built up from a β-sheet of three anti-parallel β-strands linked to a β-hairpin by a short α-helical turn [179] (Figure 6B).

The mannose-binding properties of these lectins exhibiting the CVN-fold account for their anti-HIV-1 activity.

## 4. The Mannosyl-Binding Specificities of Mannose-Binding Lectins

The high-resolution X-ray structures for a series of complexes between the isolectins LoLI and LoLII from the Cyprus vetch (*Lathyrus ochrus*) and various *N*-oligosaccharides of increasing complexity including tri-, octa- and dodecasaccharides, accomplished a breakthrough by providing a new framework for understanding how plant lectins specifically accommodate sugar units of complex *N*-glycans [191,192,193]. Additional crystal structures of other Man-specific lectins in complex with *N*-oligosaccharides allowed to decipher the complexity of the carbohydrate-binding of complex glycans to plant and fungal lectins at the molecular level (Table 3).

Depending on the molecular complexity of the recognized carbohydrates, two types of closely interlinked carbohydrate-binding specificities can occur at the carbohydrate-binding site of plant and fungal lectins:

1. A monosaccharide-binding specificity, allowing the lectin to specifically recognize a simple sugar, e.g., mannose Man, and its derivatives, e.g., α-methylmannoside. This type of monosaccharide recognition by lectins corresponds to the so-called “broad sugar-binding specificity” of lectins, which relies on the occurrence of a monosaccharide-binding pocket within the carbohydrate-binding site.

2. An oligosaccharide-binding specificity, which consists of the simultaneous accommodation of several sugar units of a complex *N*-glycan, e.g., high-mannose glycans, also known as the “fine sugar-binding specificity” of the lectins. This type of oligosaccharide recognition involves most of the surface of the carbohydrate-binding site, including the monosaccharide-binding site. 

The monosaccharide-binding site is part of a more extended oligosaccharide-binding site. In physiological conditions, however, plant and fungal lectins are almost always involved in the recognition of complex glycans, rather than simple sugars, simply because the amount of free monosaccharides in cells and tissues is very low. The binding of plant and fungal lectins to mannose was first observed in hapten inhibition experiments, by introducing free mannose or mannose derivatives to prevent or reverse the in vitro interaction between lectins and red blood cells or complex glycans. Obviously, the affinity of mannose-specific lectins for simple sugars, e.g., for Man or Man derivatives, is far weaker compared to the affinity measured for more complex glycans, e.g., for complex *N*-glycans or high-mannose type glycans (Table 4) [10,225].

### 4.1. The Mannose-Binding Specificity

The recognition and binding of simple sugars by lectins occurs through non covalent interactions occurring between some hydroxyls of the sugar ring and a few, essentially polar, amino acid residues forming a shallow depression at the lectin surface, the so-called monosaccharide-binding site. Usually, most of these interactions consist of hydrogen bonds (H-bond) often associated to a hydrophobic stacking of the pyranose ring of the sugar to the phenolic ring of an aromatic residue such as Phe (F), Tyr (Y), or Trp (W), located in the close vicinity of the monosaccharide-binding cavity. Acidic residues like Asp (D) and Glu (E), often participate in the interaction with simple sugars, thus attributing a more or less pronounced electronegative character to the monosaccharide-binding site. Both acidic residues Asp and Glu, play a key role in the binding of simple sugars due to their capacity to create multiple H-bonds with the hydroxyls emerging from the sugar ring.

**Figure 7:**
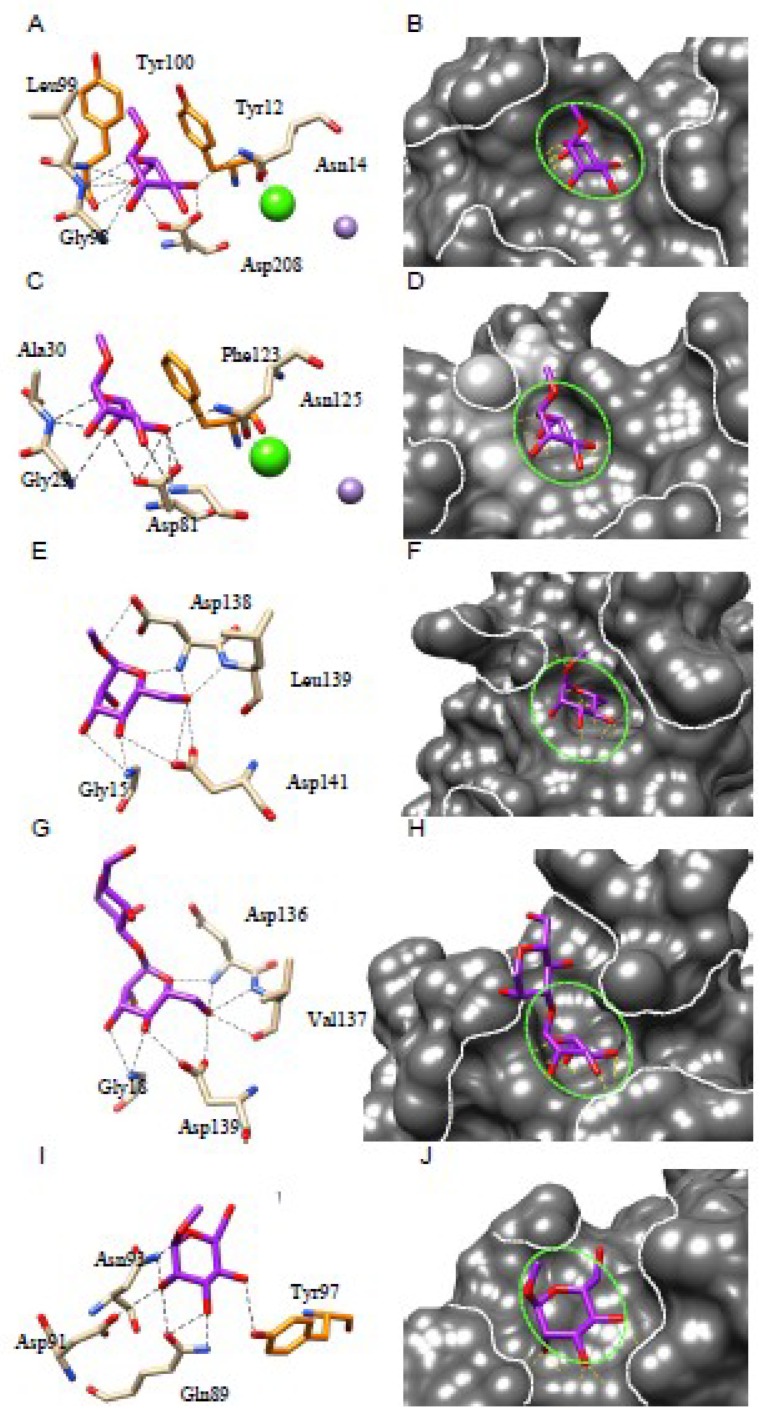
(**A**,**B**). ConA from *Canavalia ensiformis* in complex with α-methylmannoside (PDB code 5CNA). (**C**,**D**). Isolectin LoLI from *Lathyrus ochrus* in complex with Man (PDB code 1LOB). (**E**,**F**). Artocarpin from *Artocarpus integrifolia* in complex with α-mthylmannoside (PDB code 1J4U). (**G**,**H**). Heltuba from *Helianthus tuberosus* in complex with Manα1,3Man (PDB code 1C3M). (**I**,**J**). Third Man-binding site of GNA from *Galanthus nivalis* in complex with α-methylmannoside (PDB code 1MSA). Hydrogen bonds connecting the monosaccharides to the amino acid residues forming the monosaccharide-binding site are represented by black dashed lines. Aromatic residues participating in stacking interactions with the sugar rings are colored orange. The molecular surface of the lectins is colored dark grey and their extended oligosaccharide-binding areas are delineated by white dashed lines. The shallow depression corresponding to the monosaccharide-binding site that accommodates simple sugars is delineated by a green dashed line. The green and violet spheres correspond to the Ca^2+^ and Mn^2+^ ions, that have a stabilizing effect on the carbohydrate-binding site.

Detailed structural information is available for the binding of α-D-mannose (Man) to the monosaccharide-binding site of Man-specific legume lectins including Con A [202], LoLI isolectin from *Lathyrus ochrus* [206], favin from the broad bean *Vicia faba* [63], pea lectin PsA [209] and PAL from *Pterocarpus angolensis* [210]. A very similar binding scheme occurs for both the two-chain (LolLI, favin, PsA) and single-chain (Con A) lectins: a few amino acid residues located on three distinct loops exposed at the top of the dome-shaped lectin protomer, form a shallow depression which accommodates the Man ligand via a network of hydrogen bonds connected to the O3, O4, O5 and O6 atoms of the sugar. An acidic residue (Asp208 of Con A, Asp81 of LoLI and PsA), which also participates in the binding of a Ca^2+^ ion located in the close vicinity of the binding site, plays a key role in ligand binding. An additional stacking interaction between the pyranose ring of Man and one (Phe123 of LoLI) or two (Tyr12 and Tyr100 of Con A) aromatic residues located in the vicinity of the monosaccharide-binding site, reinforces anchorage of the sugar to the binding site (Figure 7A–D). A few water molecules also participate in the binding of Man to the monosaccharide-binding site of the lectins. Very similar binding observations were reported for the binding of Man or α-methyl-D-mannoside (MeMan) to other *Canavalia* [20,21,25,27] and *Dioclea* lectins [34,35,36,37,38,39,40,41,42,43,44], *Parkia biglobosa* (PDB code 4MQ0) and *Cymosema roseum* (PDB code 4MYE) Man-specific lectins from the Brasilian flora.

The accommodation of Man by artocarpin, a Man-specific jacalin-related lectin, shows a very similar network of 9 H-bonds between four amino acid residues (Gly15, Asp138, Leu139, Asp141) located at the top of the β-prism protomer, and the O1, O3, O4, O5, and O6 atoms of the sugar (Figure 7E,F). No stacking interactions occur between the aromatic residues of the monosaccharide-binding site and the sugar. In addition, jacalin, another member of the jacalin-related lectins, offers an interesting example of sugar-binding promiscuity because this Gal-specific lectins also interacts, albeit with lower affinity, with other simple sugars like Man, Glc and GalNAc via a very similar H-bond network [77]. Another Man-specific lectin with a β-prism architecture, Heltuba of *Helianthus tuberosus*, also accommodates Man through a very similar network of H-bonds between four amino acid residues (Gly18, Asp136, Val137, Asp139), which form the monosaccharide-binding site also located at the top of the β-prism protomer, and the O3, O4, O5 and O6 atoms of the sugar (Figure 7G,H).

The recognition of Man by GNA, the Man-specific snowdrop (*Galanthus nivalis*) lectin, and other monocot Man-binding lectins harboring a similar β-prism architecture (a β-prism in which the strands composing the β-sheet are arranged perpendicularly to the axis of the prism), exhibits a different mode of binding due to the fact that three out of eight H-bonds connecting the Gln89, Asp91, Asn93 and Tyr97 residues from the 3rd mannose-binding site to the O2, O3, O4, and O6 atoms of Man, are connected to the axial O2 atom (Figure 5I,J). Residue Tyr97 also provides a stacking interaction with one face of the Man pyranose ring. An additional hydrophobic interaction with Val95, another residue of the consensus sequence stretch QXDXNXVXY of the monosaccharide-binding binding site, reinforces the anchorage of Man to the binding site.

**Figure 8:**
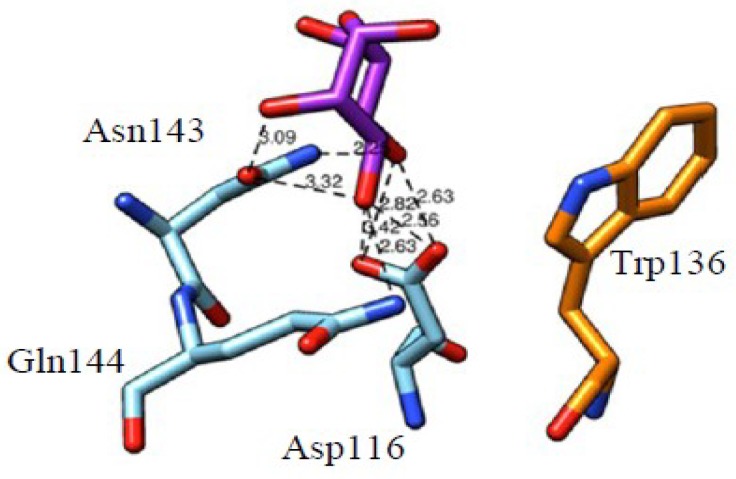
Docking of αMeMan to the monosaccharide-binding site of the active sub-domain III of OsEULS3. Hydrogen bonds connecting Man to the amino acid residues forming the monosaccharide-binding site are shown by black dashed lines and distances are indicated in Å. The aromatic Trp136 residue participating in stacking interactions with the sugar ring is colored orange.

Molecular modeling and in silico docking suggest that other nucleocytoplasmic EUL domain-containing lectins from rice (*Oryza sativa*) and *Arabidopsis* with a β-prism architecture, also interact with mannose via a very similar network of H-bonds and stacking interactions with aromatic amino acid residues located in the close vicinity of the monosaccharide-binding site (Figure 8) [170]. However, some promiscuity was shown to occur at the monosaccharide-binding site of the EUL-lectins, which in addition to high mannose *N*-glycans also recognize blood group B related structures and galactosylated epitopes [226].

### 4.2. The Oligosaccharide-Binding Specificity

Although the monosaccharide-binding capacity of Man-specific lectins has been widely investigated, it is obvious that simple sugar residues like Man probably cannot be considered as the natural ligands for plant and fungal lectins, due to the extreme scarcity of simple sugars as free ligands occurring in living organisms, compared to other complex carbohydrates. Along this line, the affinity of Man-specific lectins for complex high-mannose *N*-glycans is much higher than that measured for free Man [10,225]. In fact, once the first crystallographic structures of complexes of Man-specific lectins with oligomannosides were solved at atomic resolution [191,192,193], it became evident that the so-called monosaccharide-binding site is in fact part of a more surface-extended oligosaccharide-binding site, comprising other amino acid residues susceptible to chemical interaction with other sugar units distinct from that recognized by the monosaccharide-binding site. Such a multiplicity of interactions readily accounts for the higher affinity of Man-specific lectins for high-mannose *N*-glycans (inhibitory activity in the mM range), compared to free Man (inhibitory activity in the µM range) [225]. In addition, depending on the degree of freedom of the different *O*-glycosidic linkage types, e.g., α1-2, α1-3, α1-4 or α1-6, occurring along the glycan chain, complex glycans can more or less fit the shape of the lectin oligosaccharide-binding site.

**Figure 9:**
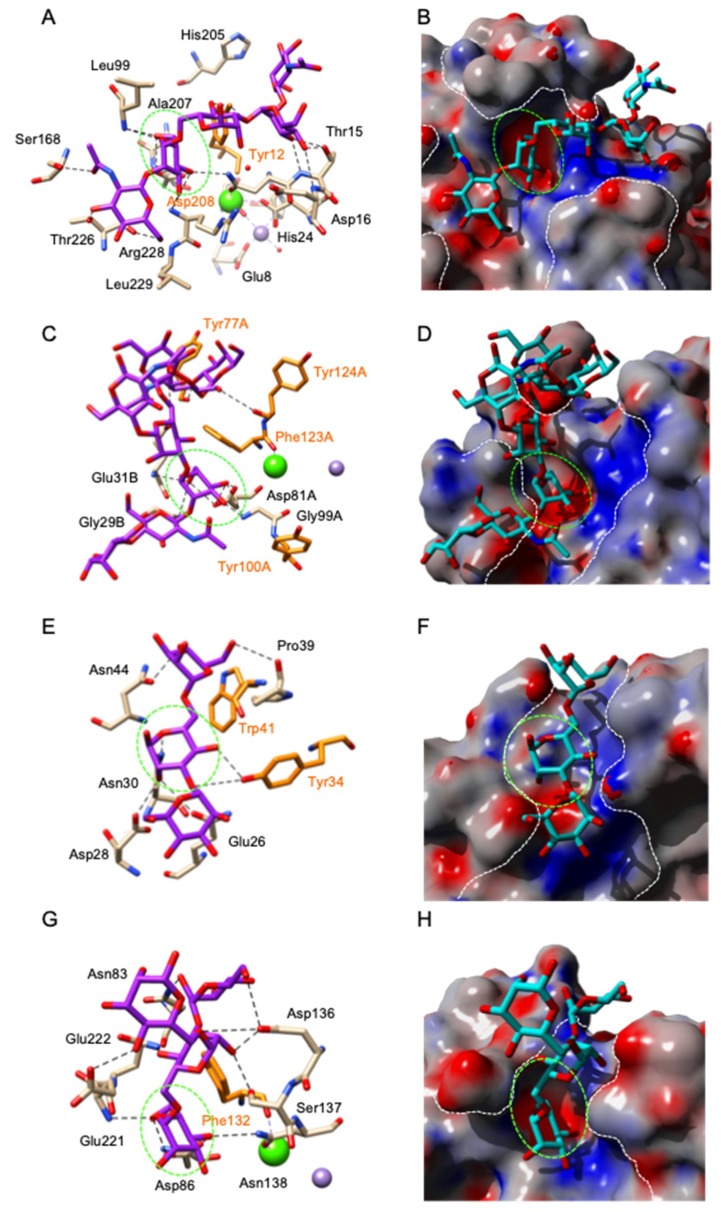
(**A**,**B**). ConA from *Canavalia ensiformis* in complex with β-D-GlcNAc-(1,2)-α-D-Man-(1,6)-[β-D-GlcNAc-(1,2)-α-D-Man-(1,6]-αD-Man (PDB code 1TEI) [196]. (**C**,**D**). Isolectin LoLII from *Lathyrus ochrus* in complex with a biantennary octasaccharide of the *N*-acetyllactosamine type from lactotransferrin (PDB code 1LOF). (**E**,**F**). GNA from *Galanthus nivalis* in complex with three mannosyl residues from a mannopentaose (PDB code 1JPC). (**G**,**H**). PAL from *Pterocarpus angolensis* in complex with a mannotetraose (PDB code 2PHF). Hydrogen bonds connecting the oligosaccharides to the amino acid residues forming the extended carbohydrate-binding site are represented by black dashed lines. Aromatic residues participating in stacking interactions with the sugar rings are colored orange. The electrostatic potentials were calculated and mapped on the molecular surface of the lectins, using YASARA. The extended oligosaccharide-binding areas are delineated by white dashed lines. The shallow depression corresponding to the monosaccharide-binding site that accommodates simple sugars, is delineated by a green dashed line.

Structural analysis of different lectin-oligosaccharide complexes (Table 5), including Con A in complex with a pentasaccharide (Figure 9A,B), isolectin LoLII from *Lathyrus ochrus* in complex with a biantennary octasaccharide of the *N*-acetyllactosamine type from lactotransferrin (Figure 9C,D), GNA in complex with a mannopentaose (Figure 9E,F), and PAL from *Pterocarpus angolensis* in complex with a mannotetraose (Figure 9G,H), show that a complex network of H-bonds, stacking and hydrophobic interactions, links several sugar units of the glycan chain to the oligosaccharide-binding site of the lectin. However, depending on the lectin, important discrepancies occur in the accommodation of sugar units. In this respect, isolectins of *Lathyrus ochrus* and other two-chain Vicieae lectins such as pea PsA and lentil LcA lectins, which differ from Con A by a higher affinity for fucosylated glycans of the *N*-acetyllactosaminic type [10,225], strongly interact with the α1,6-Fuc residue linked to the Asn-bound GlcNAc of the glycan whereas Con A does not interfere at all with the Fuc residue. Similarly, the accommodation of structurally closely-related oligomannosides by GNA (Figure 9F) and PAL (Figure 7H), illustrates how discrepancies observed in the topographical features (shape and size) of the oligosaccharide-binding site can affect the binding of complex glycans to different Man-specific lectins belonging to distinct scaffold architectures. 

**Figure 10:**
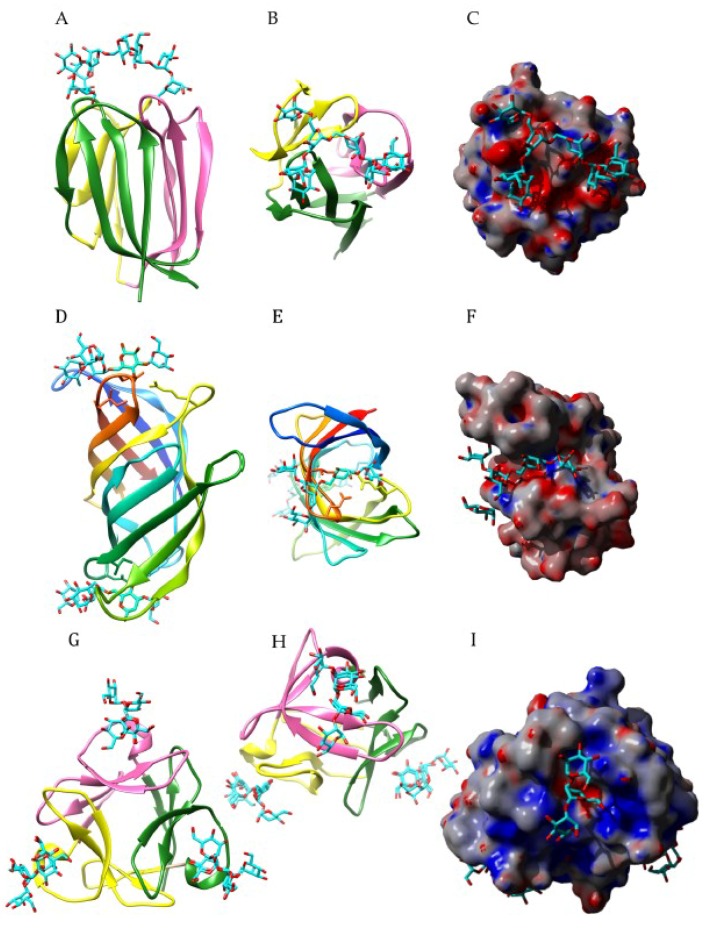
Structural diversity of the mannose-binding lectins. (**A**–**C**). Ribbon diagrams (**A** lateral view, **B** upper view) and surface electrostatic potentials (**C**) of griffthsin in complex with a high-mannose branched glycan (colored cyan) (PDB code 3LL2), showing the β-prism organization of the lectin. Note the electronegatively charged character (colored red) of the Man-binding pockets at the upper face of the β-prism. (**D**–**F**). Ribbon diagrams (**D** lateral view, **E** upper view) and surface electrostatic potentials (**F**) of actinohivin in complex with a high-mannose branched glycan (colored cyan) (PDB code 3S5X), showing the β-trefoil (β-prism II) organization of the lectin. Note the electronegatively (colored red) and electropositively (colored blue) charged character of the Man-binding pockets at the edges of the β-trefoil. (**G**–**I**). Ribbon diagrams (**G** lateral view, **H** upper view) and surface electrostatic potentials (**I**) of actinohivin in complex with α-1,2-mannotriose (colored cyan) (PDB code 4P6A), showing the organization of the lectin. Note the electronegatively charged character (colored red) of the Man-binding pockets.

Investigations on the oligosaccharide-binding specificity of Man-specific bacterial lectins, showed a highly similar binding scheme associated to the recognition of oligomannosides and complex high-mannose *N*-glycans. However, depending both on the extent of the glycan chain and the shape and size of the oligosaccharide-binding site in the lectin monomer, which possesses a β-prism- (griffthsin) or a β-barrel-architecture (actinohivin), rather distinct accommodation schemes were observed for these lectins (Figure 10) [227]. The oligosaccharide-binding sites of griffthsin and actinohivin readily differ by the shape, the size and the discrete distribution of charged residues that account for the differences observed in the accommodation of oligomannosides and high-mannose branched glycans by the lectins. Similar to plant lectins, the monosaccharide-binding pocket occupies a pivotal position at the centre of the binding site and fully participates in the binding of the complex glycans.

Obviously, the binding of complex glycan chains to lectins is a highly complex interaction process due to the extreme variability observed in the topographical features of the oligosaccharide-binding site of lectins, associated to the extreme diversity of the recognized glycan structures. Hopefully, the recent developments in glycan array technology [228], and the improvement of frontal affinity chromatography [229], offer new important tools for deciphering the biomolecular interactions between plant lectins and the large panel of complex glycans.

## 5. Functions of Mannose-Specific Lectins

The Man-specific lectins present in seeds or storage organs (bulbs, rhizomes) of plants, are abundant proteins with a dual role as storage proteins and defense proteins [230,231]. In contrast, Man-specific lectins occurring in the nucleus or in the cytoplasmic compartment are usually synthesized at low levels. Since lectin concentrations are higher after exposure of the plant to e.g., salt or drought stress, or pathogen infections these stress inducible lectins are involved in plant immunity and can help the plant to cope with environmental stresses [1,231].

### 5.1. Insecticidal Activity

A large group of GNA-related lectins have been investigated with respect to their insecticidal properties. The interest in the monocot Man-specific lectins was triggered because these lectins showed toxicity towards aphid pests responsible for serious crop damage (Table 6). The expression of GNA and other monocot lectins in various transgenic plants conferred enhanced resistance to sap-sucking aphid predators. In addition, these lectins often caused a higher larval mortality and retardation in larval development. Similarly legume lectins such as Con A were investigated for their deleterious effects on aphid growth and development. At present, the mechanism of entomotoxicity still remain poorly understood and most probably depends on diverse, complementary mechanisms [232]. 

The detrimental effects of Man-specific lectins on aphids relies on their ability to recognize and bind high-mannose glycan receptors present in the peritrophic membrane and the underlying midgut epithelium. Receptors proteins for the monocot Man-specific lectins (ACA of *Allium cepa*, *Diefenbachia sequina* lectin, CEA of *Colocacia esculenta*, AMA of *Arum maculatum*) have been identified in brush border membrane vesicles of the midgut [233], and two major receptors for AMA of 40 kDa and 35 kDa, respectively, were detected in the brush border membrane vesicles of the aphids *Lipaphis erysimi* and *Aphis craccivora* [234]. A major binding protein for Con A was identified as a membrane-bound aminopeptidase of 130 kDa, in the pea aphid *Acyrthosiphon pisum* [235]. Two other abundant membrane-associated proteins, an alanyl aminopeptidase N and a sucrase, have also been postulated as possible receptors in *Acyrthosiphon pisum*, for both garlic lectins ASAI and ASAII [236]. Interestingly, a putative glycosylated receptor of 37 kDa identified in the mushroom *Rhizoctonia solani* cross-reacted with the homodimeric *Allium sativum* leaf lectin and the interaction, which depends on the oligomeric assembly of the lectin, was specifically inhibited by addition of mannose [237]. Binding partners of CEA, the *Colocasia esculenta* Man-specific lectin, were identified as ATPase and ATP synthase in *Bemisia tabaci*, and ATP synthase, HSP70 and clathrin heavy chain in *Lipaphis erysimi* [238]. The dietary ingestion of Con A resulted in a marked decrease of the α-glucosidase and alkaline phosphatase activity in the bird cherry-oat aphid *Rhopalosiphum padi* [232]. Taken together, these results argue for multiple so-called lectin “receptors” occurring in aphid pests.

Beyond the alterations resulting from the binding of Con A to the midgut epithelial cells in the pea aphid *Acyrthosiphon pisum*, e.g., the cellular swelling of epithelial cells associated with hypersecretion [259], other systemic effects of Con A like DNA damage accompanied with an increase in caspase 3 activity in the gut tissues, were observed in the aphid *Rhopalosiphum padi* fed with a Con A-containing diet [232]. A similar entomotoxic effect accompagnied by DNA fragmentation and caspase-3-dependent apoptosis, was observed in *Acyrthosiphon pisum* fed with a diet containing the lectin SNA-I from *Sambucus nigra*, a chimerolectin corresponding to a type II RIP with a carbohydrate-binding B chain displaying sialic acid-binding specificity [260]. Finally, in addition to the direct effect of aphicidal lectins on death of gut epithelial cells, an effect on the feeding behavior has been invoked to account for the entomotoxicity of plant lectins towards aphid pests [232].

### 5.2. Resistance to Abiotic (and Biotic) Stresses

Although most lectins studied at present are constitutively expressed in plant tissues, some lectins are considered as stress inducible proteins. The discovery of Nictaba, a tobacco (*Nicotiana tabacum*) lectin which is synthesized in response to a jasmonic acid (JA) treatment, and insect herbivory, and accumulates in the cytosol and the nucleus of leaf cells [5], shed a new light on plant lectins and allowed the development of very new concepts on the role(s) of plant lectins [4,231,261,262].

Besides Nictaba, which appears as the prototype of a group of closely related lectins [263,264], other groups of stress inducible lectins in the nucleocytoplasmic compartment have been identified, such as the *Euonymus europaeus* EUL-related lectins [170,265] and the group of mannose-binding jacalin-related lectins [221,266]. Among the stress inducible cytoplasmic/nuclear lectins identified so far, Nictaba belonging to the Nictaba-related lectins [267], Orysata belonging to the jacalin-related lectins [268], and OrysaEULD1A belonging to the EUL-related lectins [269], all readily interact with high-mannose glycan structures (Table 7). Accordingly, they participate as signaling molecules in the plant response to stress conditions [270]. In this respect, a member of the EUL-related lectin family, AthEULS3 is involved in abscissic acid (ABA)-induced stomatal closure [271]. Overexpression of the Nictaba-like lectin genes *Gm*LLL1 and *Gm*NLL2 from soybean in *Arabidopsis thaliana*, was reported to confer tolerance to *Pseudomonas syringae* infection, aphid (*Aphis glycines*) infestation and salt stress [272]. Similarly, the involvement of Nictaba homologs from *Arabidopsis thaliana* in the plant stress response was recently demonstrated [273].

Recent studies for lectin sequences in several complete plant genomes reported the occurrence of chimeric proteins composed of a lectin domain with known Man-binding specificity, such as e.g., the GNA-like domain or legume lectin domain, linked to an intracellular kinase domain through a transmembrane linker domain. These lectin-receptor-like kinases (LecRLK), play a role in the signaling cascades triggered in response to biotic and abiotic stress [270].

## 6. Medical Applications for the Mannose-Specific Lectins

So far, medical applications of Man-specific lectins, have been developed in two domains: (1) as inhibitors of the entry of HIV-1 into CD4+ T-lymphocytes and, (2) as anticancer drugs for the chemotherapeutic treatment of cancers.

### 6.1. Mannose-Specific Lectins as Immunomodulators

**Figure 11:**
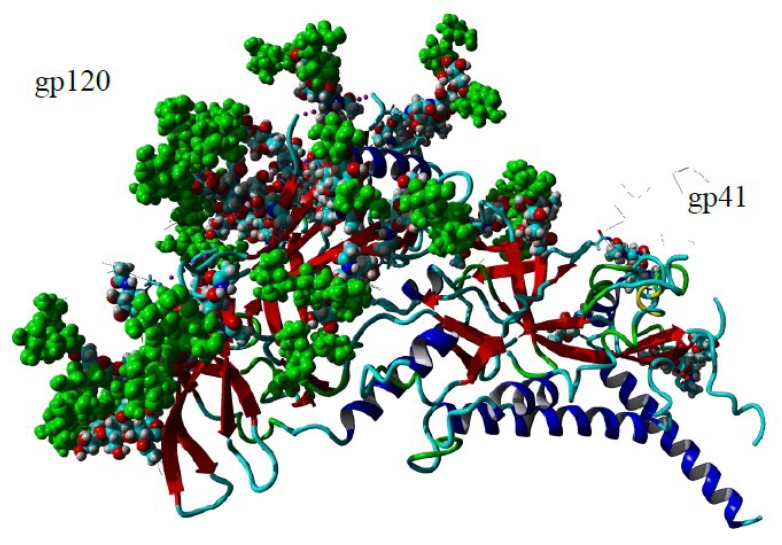
Three-dimensional structure of the highly-mannosylated gp120 molecule associated to the *O*-glycosylated gp41 molecule (PDB code 5FYK). Surface-exposed Man residues of high-mannose *N*-glycoproteins decorating gp120 are colored green. *O*-glycans of gp41 are colored blue.

**Figure 12:**
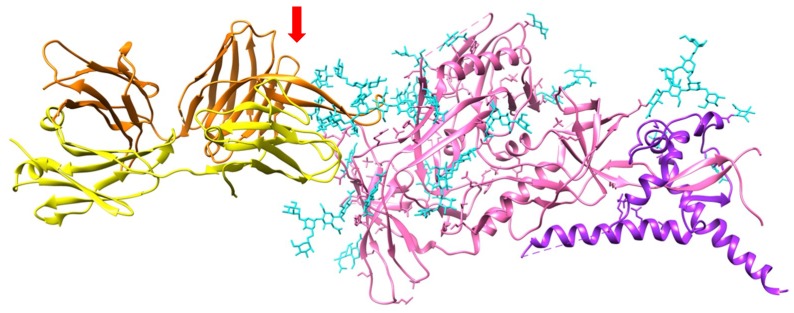
Three-dimensional structure of the gp120-gp41 tandem complexed to a CD4 molecule (PDB code 47VP). Gp120, gp41 and CD4 are colored pink, purple, and orange/yellow, respectively. The high-mannose *N*-glycan chains decorating gp120 are represented in cyan colored sticks. The carbohydrate binding agents (red arrow) specifically recognize some of the high-mannose *N*-glycans exposed at the surface of gp120, thus preventing the recognition of gp120 by the CD4 molecule of the CD4+ T lymphocytes. In fact, the association of three gp120-gp41 tandems forms the HIV-1-envelope spike, which facilitates the HIV-1 entry. The Env spike consists of a transmembrane trimer of gp41 associated to an extracellular trimer of gp120 offering exposed high-mannose glycans to the CD4 recognition process.

Soon after the identification of high-mannose *N*-glycans decorating the gp120 protein of HIV-1 (Figure 11 and Figure 12) [274], many studies focused on the use of mannose-specific lectins from bacteria, mushrooms and plants as tools to decipher the importance of the high-mannose moiety of gp120 for the recognition by the CD4+ T-lymphocytes as well as for preventing the virion infectivity of HIV toward the host cells in vitro [275,276,277,278,279,280]. Two classes of mannose-specific lectins from the Vicieae tribe and the GNA-related lectins, were particularly investigated with respect to their blocking capacity (Table 8).

Mannose-binding lectins of bacterial origin were identified as potent HIV-1-inactivating proteins through their specific binding to the envelope glycoprotein gp120. Studies have been performed for actinohivin [180], cyanovirin-N [281,282], MVL from *Microcystis viridis* [283], OAA from *Oscillatoria agardhii* [284] and the lectin from *Scytonema varium* [285].

Investigating the surface carbohydrates of gp120 showed that resistance of cyanovirin N- and Con A-resistant HIV-1 strains highly depends on mutations that have eliminated *N*-linked glycans on gp120 [310]. In general, the number of *N*-glycan deletions in gp120 correlated with the level of phenotypic resistance to cyanovirin of the mutated VIH-1A strains [311]. A similar observation was previously reported in a series of mutant HIV-1 isolates resistant to GNA (*Galanthus nivalis*) and HHA (*Hippeastrum* sp. hybrid) lectins, showing that the major amino acid mutations occur at several putative *N*-glycosylation sites NXT and NXS, and especially, at the ultimate T or S residues [312]. Removal of two high-mannose *N*-glycans in gp120 resulted in an enhanced resistance of HIV-1 to griffithsin [308]. In fact, the association of three gp120-gp41 forming the HIV1-envelope spike, will be necessary for the recognition by CD4+ T-lymphocytes [313].

Long term exposure of HIV to cyanovirin or monocot mannose-binding lectins like GNA, HHA and NPA, was shown to progressively result in the deletions of some *N*-glycan chains decorating the envelope gp120, in an attempt of the retrovirus to diminish the drug pressure and acquire resistance against the carbohydrate-binding agents [314,315,316]. In addition, the associated treatment of mutant virus strains by 1-deoxymannojirimycin, a potent inhibitor of the α(1,2)-mannosidase, strongly enhanced the suppressive effect of carbohydrate-binding agents on VIH-1 replication [317]. A similar synergistic effect was also observed when combining two carbohydrate-binding agents that recognize distinct *N*-glycan structures decorating the gp120 [318].

Additionaly, a 13 kDa monomeric mannose-binding lectin from edible chive (*Allium tuberosum*), exhibited pronounced inhibitory activity against the HIV-1 reverse transcriptase, a key enzyme in the replication of the HIV-1 genome [319]. However, other lectins with different carbohydrate-binding specificities like PHA from *Phaseolus vulgaris*, RCA from *Ricinus communis* and ABA from the mushroom *Agaricus bisporus*, also exhibited a similar inhibitory activity against the HIV-1 reverse transcriptase [320].

Moreover, the bacterial carbohydrate-binding agents cyanovirin-N, griffithsin and scytovirin, also inhibit the syncytium formation in different HIV-1 infected and uninfected cell lines by preventing the DC-SIGN receptor-directed HIV-1 capture by monocyte-derived dendritic cells (DCs), and subsequent transmission to CD4+ T-lymphocytes [321,322,323,324].

Other lectins with very different carbohydrate-binding specificities like the Gal/GalNAc-specific jacalin from *Artocarpus integrifolia* and the GlcNAc-specific Nictaba from *Nicotiana tabacum*, are also potent inhibitors for the HIV-1 infection of CD4+ T lymphocytes [325,326]. In fact, both lectins exhibit some mannose-binding promiscuity as shown from X-ray crystallographic experiments for jacalin [77,78], and glycan array experiments for Nictaba [267], respectively.

### 6.2. Mannose-Specific Lectins as Cancer Biomarkers and Anti-Cancer Drugs

The ability of mannose-specific lectins to distinguish between normal and diseased cancer cells through the selective recognition of the altered hypermannosylation *N*-glycans associated to various tumor cell transformations, has led to the application of mannose-binding lectins as potential biomarkers for the detection and the follow up of different tumor cells. In this respect, a variety of legume Man-specific legume lectins and GNA-like lectins were deeply investigated (Table 9).

The recognition of altered *N*-glycans covering the cancer cells by lectins resulted in programmed cell death through targeting of different apoptotic and autophagic pathways. However, the effects of plant lectins on programmed cell death of cancer cells is not limited to Man-specific lectins since, other plant lectins with distinct carbohydrate-binding specificities, e.g., T/Tn-specific lectins, also interfere with other altered *O*-glycans covering tumor cells to exert their cytotoxic effects [327].

Plant and fungal lectins affect both apoptosis and autophagy in cancer cells by modulating diverse signaling pathways associated to various pro-apoptotic gene families including, but not exclusively, the Bcl-2 family, caspase family, ROS-p38-p53, P73-Foxo1a-Bim apoptosis, PI3K/Akt, ERK, BNIP3-mediated mitochondrial autophagy, Ras-Raf family and ATG family [328,329,330] (Table 9). However, depending on both the lectins and the type of targeted cancer cells, some discrepancies occur with respect to the apoptotic and autophagic pathways leading to the programmed cell death.

Following to these cytotoxic effects on cancer cells, some therapeutic applications have been considered, essentially for the Man-specific legume lectins (Con A) and the GNA-related lectins (*Polygonatum cyrtonema*) [359,360,361]. To date, however, the use of plant lectins as targeting tools for therapeutic applications has rarely been used [362].

## 7. Biomedical Perspectives for Mannose-Specific Lectins

Obviously, Man-specific lectins from plants, algae and fungi are interesting probes to target the altered hypermannosylated *N*-glycan expressed at the surface of malignant cells. Our knowledge on the fine carbohydrate-binding specificity of plant and fungal lectins revealed the extreme versatility of the Man-specific lectins to specifically recognize discrete/subtle differences in the expression of altered glycans by tumor cells. Depending on the discrepancies observed in both the shape and size of their extended carbohydrate-binding site, the affinity towards high-mannose *N*-glycans and their chemical substitutions such as sialylation or sulfation varies widely from one lectin to another. The ability of Man-specific lectins to accommodate large mannosylated chains to the extended carbohydrate-binding site via a complex network of hydrogen bonds and hydrophobic interactions, readily accounts for such versatility. Compared to monoclonal antibodies used as standard probes for the detection of the glycan aberrations occurring at the cancer cell surface, plant and fungal Man-specific lectins are a complementary and equally powerful tool for the recognition of high-mannose *N*-glycans [362]. Besides the high-mannose *N*-glycan recognition, Man-specific lectins can exert cytotoxic effects on the targeted cancer cells. They induce apoptotic and autophagic death through modulation of different signaling pathways in cancer cells. These encouraging results suggest the potential use of carefully selected Man-specific lectins for the treatment of cancers [361,362,363].

Besides their cytotoxic effects detrimental for cancer cells, Man-specific lectins have been proven to act as valuable anti-HIV drugs in vitro and in vivo. The Man-specific lectins from plant, fungal and bacterial origin constitute an important class of HIV entry inhibitors by virtue of their capacity to specifically recognize and bind the oligomannoside chains decorating the evelope gp120 of HIV [363]. Moreover, a long-term exposure of HIV to plant and fungal lectins results in deletions in some of the *N*-glycan chains of gp120, as an attempt of the virus to escape drug pressure, that improves the antiviral activity of these carbohydrate-binding agents [318]. However, the therapeutic use of Man-specific lectins still suffers from several limitations dealing with their high manufacturing costs, formulation and potential mitogenicity, as stated in [364]. In spite of these limitations, encouraging results have been reported using lectins via topical mucosa administration [324].

## Figures and Tables

**Table 1:** Overview of plant, algae and fungi lectins with a mannosyl-binding specificity (β-sandwich: βs, β-prism: βp, n.d.: not determined).

Plant, Alga, Mushroom Family	Plant, Alga, Mushroom Species	Lectin	Structural Scaffold	Oligomer	Ref.
Pteridophyta	*Phlebodium aureum*	PAL	β barrel	2	[12]
Gymnosperms	*Araucaria brasiliensis*	Lectin I	n.d.	10	[13]
		Lectin 2	n.d.	6	
	*Gingko biloba*	Gnk2	α β	1	[14]
	*Cycas revoluta*	CRLL	β-prism	2	[15,16]
Fabaceae	*Bowringia mildbraedii*	BMA	β-sandwich	2/4	[17]
	*Cajanus cajan*	CcL	βs	2	[18]
	*Camptosema pedicellatum*	CPL	βs	4	[19]
	*Canavalia boliviana*	ConBo	βs	4	[20]
	*Canavalia bonariensis*	CaBo	βs	4	[21]
	*Canavalia brasiliensis*	ConBr	βs	4	[22]
	*Canavalia ensiformis*	ConA	βs	4	[23]
	*Canavalia gladiata*	CGL	βs	4	[24]
	*Canavalia grandiflora*	ConGF	βs	4	[25]
	*Canavalia maritima*	ConM	βs	4	[26]
	*Canavalia virosa*	ConV	βs	4	[27]
	*Centrolobium microchaete*	CML	βs	4	[28]
	*Centrolobium tomentosum*	CTL	βs	4	[29]
	*Cladrastis lutea*	CLAI,II	βs	4	[30]
	*Cratylia floribunda*	CFL	βs	2/4	[31]
	*Cratylia mollis*	CRAMOLL	βs	2/4	[32]
	*Cymbosema roseum*	CRLI	βs	4	[33]
	*Dioclea grandiflora*	DGL	βs	4	[34,35]
	*Dioclea guianensis*	Dguia	βs	4	[36]
	*Dioclea lasiocarpa*	DLL	βs	4	[37]
	*Dioclea lasiophylla*	DlyL	βs	4	[38]
	*Dioclea reflexa*	DrfL	βs	4	[39]
	*Dioclea rostrata*	DRL	βs	4	[40]
	*Dioclea sclerocarpa*	DSL	βs	4	[41]
	*Dioclea violacea*	DVL	βs	4	[42]
	*Dioclea virgata*	DvirL	βs	4	[43]
	*Dioclea wilsonii*	DwL	βs	4	[44]
	*Lathyrus aphaca*	LaphL	βs	2	[45]
	*Lathyrus articulatus*	LarL	βs	2	[45]
	*Lathyrus cicera*	LcL	βs	2	[45]
	*Lathyrus hirsutus*	LhL	βs	2	[46]
	*Lathyrus nissolia*	LnL	βs	1	[47]
	*Lathyrus ochrus*	LoL	βs	2	[48]
	*Lathyrus odoratus*	LodL	βs	2	[49]
	*Lathyrus sativus*	LsL	βs	2	[50]
	*Lathyrus sphaericus*	LsphL	βs	1	[51]
	*Lathyus sylvestris*	LsiL	βs	2	[52]
	*Lathyrus tingitanus*	LtL	βs	2	[46]
	*Lens culinaris*	LcA	βs	2	[53]
	*Millettia dielsiana*	MDL	βs	2	[54]
	*Onobrychis viciifolia*		βs	n.d.	[55]
	*Pisum arvense*	PAL	βs	2	[56]
	*Pisum sativum*	PsA	βs	2	[57]
	*Pterocarpus angolensis*	PAL	βs	2	[58]
	*Sophora flavescens*	SFL	βs	2	[59]
	*Trigonella foenumgraecum*		βs	n.d.	[60]
	*Vicia cracca*		βs	2	[61]
	*Vicia ervilia*		βs	4	[62]
	*Vicia faba*	VfA	βs	2	[63]
	*Vicia sativa*		βs	2	[64]
Mimosaceae	*Parkia biglobosa*	PBL	βs	2	[65]
	*Parkia platycephala*	PPL	βs	2	[66]
Dalbergieae	*Platypodium elegans*	nPELa	βs	2	[67]
	*Platymiscium floribundum*	PFL	βs	2	[68]
Fagaceae	*Castanea crenata*	CCA	βs	6/8	[69]
Moraceae	*Artocarpus heterophyllus*	ArtinM	β-prism	4	[70,71]
	*Artocarpus incisa*	Frutapin	βp	4	[72]
	*Artocarpus integer*	CMB	βp	4	[73,74]
	*Artocarpus integrifolia*	artocarpin	βp	4	[75,76]
		jacalin	βp	4	[77,78]
	*Artocarpus lakoocha*	artocarpin	βp	4	[79]
	*Morus nigra*	Moniga-M	βp	4	[80]
Asteraceae	*Helianthus tuberosus*	Heltuba	βp	8	[81]
Brassicaceae	*Arabidopsis thaliana*	PP2-A1	βp	n.d.	[82]
Ranonculaceae	*Clematis montana*	CML	βp	2	[83]
Aloeae	*Aloe arborescens*	ALOE	βp	4	[84]
Araceae	*Arisaema lobatum*	ALA	n.d.	2+2	[85]
	*Arisaema heterophyllum*	AHA	βp	n.d	[86]
	*Arum maculatum*	AMA	βp	2+2	[87]
	*Colocasia esculenta*	CEA, tarin	βp	2+2	[88]
	*Dieffenbachia sequina*		βp	2+2	[87]
	*Lysichiton camtschatcensis*		βp	2+2	[89]
	*Pinellia ternata*	PTA	βp	2+2	[90]
	*Remusatia vivipara*	RVL	βp	2+2	[91]
	*Typhonium divaricatum*	TDL	βp	2+2	[92]
	*Xanthosoma sagittifolium*	XSL	βp	2+2	[93]
	*Zantedeschia aethiopica*	ZAA	βp	n.d.	[94]
Asparagaceae	*Ophiopogon japonicus*	OJL	βp	n.d.	[95]
	*Polygonatum cyrtonema*	PCL	βp	4	[96]
	*Polygonatum multiflorum*	PMA	βp	4	[97]
Convolvulaceae	*Polygonatum odoratum*	POL	βp	4	[98]
	*Calystegia sepium*	Calsepa	βp	2	[99]
	*Ipomoea batatas*	ipomoelin	βp	4	[100]
Alliaceae	*Allium altaicum*	AALTA	βp	2	[101]
	*Allium ascalonicum*	AAA	βp	2	[102]
	*Allium cepa*	ACA	βp	2	[103]
	*Allium porrum*	APA	βp	2	[103]
	*Allium sativum*	ASA-I/II	βp	2	[104]
	*Allium tuberosum*	ATA	βp	2	[105]
	*Allium ursinum*	AUA-I/II	βp	2	[106]
Amaryllidaceae	*Amaryllis vittata*	AVA	βp	n.d.	[107]
	*Clivia miniata*	CMA	βs	2	[108]
	*Crinum asiaticum*	CAA	βp	n.d.	[109]
	*Galanthus nivalis*	GNA	βp	4	[110]
	*Hippeastrum hybrid*	HHA	βp	2	[111]
	*Leucojum vernum*	LVL	βp	n.d.	[112]
	*Zephyranthes candida*	ZCA	βp	4	[113]
	*Zephyranthes grandiflora*	ZGA	βp	4	[114]
	*Lycoris aurea*	LAA	βp	2	[115]
	*Lycoris radiata*	LRA	βp	2	[116]
Dioscoreaceae	*Dioscorea batatas*	DB1	βp	1	[117]
	*Dioscorea bulbifera*	DBL	βp	1	[118]
Iridaceae	*Crocus sativus*	CSL	βp	n.d.	[119,120]
	*Crocus vernus*	CVA	βp	4	[121]
Liliaceae	*Aspidistra elatior*	AEL	n.d.	2	[122]
	*Narcissus pseudonarcissus*	NPA	βp	2,4	[111]
	*Narcissus tazetta*	NTL	βp	2	[123]
	*Narcissus tortifolius*	NTA	βp	n.d.	[124]
	*Tulipa hybrid*	TxLCI	βp	4	[125]
		TL-MII	βp	2	
Smilacaceae	*Smilax glabra*	SGM2	βp	3	[126]
Hyacintheae	*Scilla campanulata*	SCAman	βp	2	[127]
Musaceae	*Musa acuminata*	BanLec	βp	2	[128]
	*Musa paradisiaca*		βp	2	[129]
Pandanaceae	*Pandanus amaryllifolius*	pandanin	βp	n.d.	[130]
Orchidaceae	*Cymbidium hybridum*	CHA	βp	2	[131]
	*Dendrobium officinale*	DOA2	βp	n.d.	[132]
	*Epipactis helleborine*	EHMBP	βp	2	[131]
	*Gastrodia elata*	gastrodianine	βp	2	[133]
	*Liparis noversa*	LNL	βp	2	[95]
	*Listera ovata*	LNL	βp	2	[131]
Poaceae	*Oryza sativa*	Orysata	βp	2	[134]
Red algae	*Bryothamnion seaforthii*	BSL	n.d.	1	[135]
	*Bryothamnion triquetrum*	BTL	n.d.	1,2	[136]
	*Euchema denticulatum*	EDA	n.d.	1	[137]
	*Eucheuma serra*	ESA	n.d.	1	[138]
	*Griffithsia* sp.	griffithsin	n.d.	2	[139,140]
	*Hypnea cervicornis*	HCA	n.d.	1	[141]
	*Hypnea japonica*	HJA	n.d.	1	[9]
	*Hypnea musciformis*	HMA	n.d.	1	[142]
	*Kappaphycus alvarezii*	KAA-2	n.d.	1	[143]
	*Kappaphycus striatum*	KSA	n.d.	1	[144]
Green algae	*Boodlea coacta*	BCA	β-prism	1	[145]
	*Halimeda renschii*	HRL40-1/2	n.d.	4	[146]
Hydnangiaceae	*Laccaria bicolor*	tectonin 2	β-propeller	n.d.	[147,148]
Trichocomaceae	*Penicillium chrysogenum*	PeCL	n.d.	n.d.	[149]
Saccharomycetaceae	*Saccharomyces cerevisiae*	Flo5A	β-sandwich	2	[150]
	*Saccharomyces pasteurianus*	Flo1p	βs	4	[151]
Schizosaccharo-mycetaceae	*Schizosaccharomyces pombe*	glucosidase	βs	2	[152]
Hygrophoraceae	*Hygrophorus russula*	HRL	n.d.	4	[153]
Marasmiaceae	*Marasmus oreades*	MOA	β-prism	2	[154]
Pteridaceae	*Ceratopteris richardii*	cyanovirin	CVN-fold	1	[155]
Sordariaceae	*Neurospora crassa*	cyanovirin	CVN-fold	1	[155]
Tuberaceae	*Tuber borchii*	cyanovirin	CVN-fold	1	[155]

**Table 2:** Overview of bacterial lectins of the CVN-fold with a mannose-binding specificity.

Bacteria Family	Species	Lec Lectin	Dstructural Scscaffold	Ref.
Actinomycetaceae	*Actinomycete* sp.	actinohivin	CVN-fold	[180]
Bukholderiaceae	*Burkholderia cenocepacia*	BcLA	id.	[181]
Cyanothecaceae	*Cyanothece* sp.	Cyt-CVNH	id.	[182]
Nectriaceae	*Gibberella zeae*	Gz-CVNH	id.	[183]
Oscillatoriaceae	*Oscillatoria agardhii*	OAA	id.	[184]
Microcystaceae	*Microcystis aeruginosa*	microvirin	id.	[179]
	*Microcystis viridis*	MVL	id.	[185]
Myxococcaceae	*Myxococcus xanthus*	cyanovirin-N	id.	[186]
Nostocaceae	*Nostoc ellipsosporum*		id.	[187]
Pseudomonadaceae	*Pseudomonas fluorescens*	PFL	id.	[186]
	*Pseudomonas putida*	LLP	id.	[188]
Scytonemataceae	*Scytonema varium*		id.	[189]
Thermotogaceae	*Thermotoga maritim*	Tmcbm27	β-sandwich	[190]

**Table 3:** PDB codes of lectins from plants and fungi, complexed with simple sugars (**m**), oligomannosides (o), and complex (c) mannose-containing glycans.

Plant Species:	Lectin:	PDB Code:	Ref.
*Bowringia mildbraedii*	BMA	2FMD(o)	[194]
*Canavalia ensiformis*	ConA	1BXH(o), 1CVN(o), 1I3H(o), 1ONA(o), 1QDC(o), 1QDO(o), 1TEI(o), 1VAM(m), 5CNA(m), 5WEY(o)	[195,196,197,198,199,200,201,202,203]
*Canavalia gladiata*	CGL	2D7F(m), 2EF6(o), 2OVU(o)	[204,205]
*Canavalia maritima*	ConM	2OW4(o), 2P37(o)	[205]
*Canavalia virosa*	ConV	5F5Q(m)	[27]
*Centrolobium tomentosum*	CTL	5EYX(o), 5EYY(o)	[29]
*Cymbosema roseum*	CRLI	4MYE(m)	
*Dioclea grandiflora*	DGL	1DGL(o)	[35]
*Dioclea lasiocarpa*	DLL	5UUY(m)	[37]
*Dioclea lasiophylla*	DlyL	6CJ9(m)	[38]
*Dioclea reflexa*	DrfL	5TG3(m)	[39]
*Dioclea rostrata*	DRL	2ZBJ	[40]
*Dioclea sclerocarpa*	DSL	4NOT(m)	[41]
*Dioclea virgata*	DvirL	3RS6(m)	[43]
*Lathyrus ochrus*	LoLI	1LOA(m), 1LOB(m), 1LOF(o), 1LOG(o)	[191,192,206]
	LoLII	1LGB(c), 1LGC(c)	[193]
*Pisum arvense*	PAL	5T7P(m)	[207]
*Pisum sativum*	PsA	1BQP(m), 1RIN(o)	[208,209]
*Pterocarpus angolensis*	PAL	1Q8O(o), 1Q8P(o), 1Q8Q(o), 1Q8S(o), 1Q8V(o), 1UKG(m), 2AR6(o), 2ARB(o), 2ARE(m), 2ARX(o), 2AUY(o), 2GN3(m), 2GN7(o), 2GMM(o), 2GMP(o), 2PHF(o), 2PHR(o), 2PHT(o), 2PHU(o), 2PHW(o), 2PHX(o)	[210,211,212,213]
*Parkia biglobosa*	PBL	4MQ0(m)	
*Artocarpus incisa*	frutapin	5M6O(m)	[72]
*Artocarpus integrifolia*	artocarpin	1J4U(m), 1VBO(o), 1VBP(o)	[163,214]
	jacalin	1KUJ(m), 1WS4(m), 1WS5(m)	[77,78]
*Morus nigra*	Morniga-M	1XXR(m)	[168]
*Helianthus tuberosus*	Heltuba	1C3M(o), 1C3N(o)	[81]
*Colocasia esculenta*	tarin	5D9Z(m), 5T20(o)	[165]
*Ipomoea batatas*	ipomoelin	3R51(m),	[99]
*Calystegia sepium*	Calsepa	1OUW(m), 5AV7(o), 5XF1(o)	[98]
*Allium sativum*	ASA	1BWU(m), 1KJ1(m)	[215,216]
*Galanthus nivalis*	GNA	1JPC(o), 1MSA(m), 1NIV(o)	[217,218]
*Narcissus pseudonarcissus*	NPA	1NPL(o), 3DZW(o)	[219]
*Musa acuminata*		3MIT(m), 3MIU(o), 4PIK(o), 4PIT(o)	[128,220]
*Musa paradisiaca*		1X1V(m)	[127]
*Oryza sativa*	Orysata	5XFH(c), 5XFI(c)	[221]
**Fungal/Algal Species:**	**Lectin:**	**PDB Code:**	**Ref.**
*Griffthsia* sp.	griffthsin	2GUC(m), 2GUD(m), 2HYQ(o), 3LL2(c)	[140,222,223]
*Saccharomyces cerevisiae*	adhesin Flo1	4LHK(o), 4LHN(m)	[151]
*Saccharomyces pastorianus*	flocculin Flo5	2XJP(m), 2XJR(o), 2XJS(o), 2XJT(o), 2XJU(o)	[150]
*Schizosaccharomyces pombe*	glucosidase II	4XQM(m)	[152]
*Marasmus oreades*	cyanovirin-N	4TKC(m)	
*Actinomyces* sp.	actinohivin	4P6A(o)	[224]

**Table 4:** Minimum concentrations (mM) of various oligosaccharidic structures and glycopeptides necessary to completely inhibit red blood cells agglutination by Con A, LcA from lentil and favin (from ref. [225]).

Oligosaccharidic Structures	Con A	LcA	Favin
Man	1.25	2.5	0.625
αMan(1,3)βΜαν(1,4)GlcNAc	0.104	0.83	0.104
αΜαν(1,2)αΜαν(1,3)βMan(1,4)GlcNAc	0.026	0.21	0.105
αΜαν(1,2)αMan(1,2)αΜαν(1,3)βΜαν(1,4)GlcNAc	0.026	0.206	0.105
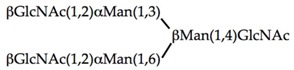	0.0003	0.157	0.31
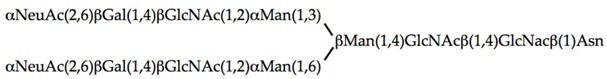	0.026	0.003	0.013
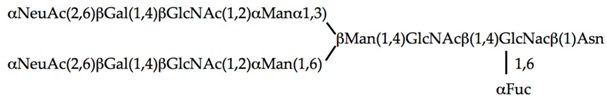	0.026	0.0004	0.0008

**Table 5:** Structure of the branched oligosaccharides complexed to Con A (PDB code 1TEI), LoLII (PDB code 1LOF), GNA (PDB code 1JPC) and PAL (PDB code 2PHF).

Oligosaccharides/Glycopeptide	Complexed to:
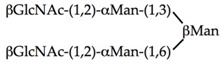	Con A
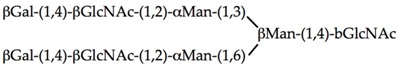	LoLII
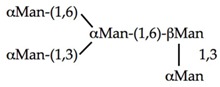	GNA
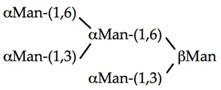	PAL

**Table 6:** List of Man-specific lectins investigated for their toxicity towards aphids (aphid predator species are indicated with an asterisk *).

Lectin class	Lectin	Aphid	Ref.
Monocot lectins	GNA (*Galanthus nivalis*)	*Aulacorthum solani*	[239]
		*Myzus persicae*	[240,241]
		*Caratovacuna lanigera*	[242]
		*Myzus persicae*	[243]
		*Rhopalosiphum maidis*	[244]
		Chrysoperla carnea* Adalia punctata*, Coccinella septempunctata*	[245]
		*Sitobium avenae, Schizaphis graminum, Rhopalosiphum padi*	[246]
	PTA (*Pinellia ternata*)	*Myzus persicae*	[247,248]
	PPA (*Pinellia pedatisecta*)	*Sitobium avenae*	[249]
	AAA (*Allium altaicum*)	*Aphis gossypii*	[100]
	ACA (*Allium cepa)*)	*Myzus persicae*	[250]
	ASA (*Allium sativum*)	*Myzus persicae*	[251]
		*Aphis craccivora*	[252]
		*Myzus nicotianae*	[253]
		*Acyrthosiphon pisum*	[236]
	AHA (*Arisaema heterophyllum*)	*Myzus persicae*	[254]
	MDA (*Monstera deliciosa*)	*Myzus persicae*	[255]
	Orysata (*Oryza sativa*)	*Acyrthosiphon pisum, Myzus persicae*	[256]
	*Dieffenbachia sequina*	*Aphis craccivora, Lipaphis erysimi*	[233]
	CEA (*Colocacia esculenta*)	*Lipaphis erysimi, Aphis craccivora*	[233]
	AMA (*Arum maculatum*)	*Aphis craccivora, Lipaphis erysimi*	[234]
	ZGA (*Zephyranthes grandiflora*)	*Myzus nicotianae*	[257]
Legume lectins	Con A (*Canavalia ensiformis*)	*Acyrthosiphon pisum*	[235]
		*Rhopalosiphum padi*	[232]
β-prism lectins	HTA (*Helianthus tuberosus*)	*Myzus persicae*	[258]
Fungal lectins	PeCl (*Penicillium chrysogenum*)	*Myzus persicae*	[149]

**Table 7:** List of the stress inducible, nucleocytoplasmic lectin families identified in plants.

Lectin Families	Carbohydrate-Binding Specificity
Jacalin-related lectin family	High-mannose *N*-glycans
EUL-related lectin family	Galactosides, high-mannose *N*-glycans
Nictaba-related lectin family	Chitooligosaccharides, recognition of the (GlcNAc)_2_-Man_3_ core of high-mannose *N*-glycans and complex glycans

**Table 8:** List of the mannose-specific lectins inhibiting HIV infection by binding to the viral gp120 envelope protein.

Lectin Class	Lectin	Ref.
Vicieae lectins	Con A (*Canavalia ensiformis*)	[276,286,287,288,289,290,291,292,293,294]
	LcA (*Lens culinaris*)	[280]
	LoLI (*Lathyrus ochrus*)	[280]
	PsA (*Pisum sativum*)	[280,295]
Monocot lectins	CHA (*Cymbidium hybrid*)	[280,296]
	EHA (*Epipactis helleborine*)	[280,296]
	GNA (*Galanthus nivalis*)	[280,297,298,299,300,301,302]
	HHA (*Hippeastrum hybrid*)	[280,297,302]
	LOA (*Listera ovata*)	[280,297]
	NPA (*Narcissus pseudonarcissus*)	[280,297,303]
	NTA (*Narcissus tazetta*)	[304]
	*Narcissus confusus, N. leonensis* and *N. tortifolius*	[124]
	PCL (*Polygonatum cytonema*)	[96,305]
Convolvulaceae	Calsepa (*Calystegia sepium*)	[280]
Urticaceae	UDA (*Urtica dioica*)	[296]
Araceae	RVL (*Remusatia vivipara*)	[91]
Musaceae	BanLec (Musa acuminata)	[306]
Poaceae	GNAmaize (*Zea mays*)	[307]
Asteraceae	Heltuba (*Helianthus tuberosus)*	[280]
Red algae	Griffithsin (*Griffithsia* sp.)	[139,308]
Green algae	KAA (*Kappaphycus alvarezii*)	[309]
	BCA (*Boodlea coacta*)	[143]

**Table 9:** Cytotoxic effects of Man-specific lectins on cancer cells (reported during the last decade).

Lectin	Cancer Cell	Apoptosis	Autophagy	Ref.
Cabo (*Canavalia bonariensis*)	glioma		+	[21]
PsA (*Pisum sativum*)	colorectal cancer	+		[331]
	Erlich acites carcinoma	+		[332]
MOSL (*Moringa oleifera*)	Erlich acites carcinoma,	+		[333]
	murine malanoma	+		[334]
DLasiL (*Dioclea lasiocarpa*)	glioma	+		[37]
	ovarian, lung, brestbreast, prostate carcinoma		+	[335]
POL (*Polygonatum odoratum*)	melanoma	+	+	[336]
	lung adenocarcinoma	+	+	[337]
	breast cancer	+	+	[338]
	lung cancer (non-small cells)	+		[339]
	melanoma	+		[98]
	murine fibrosarcoma	+		[340]
*Hyacinthus* sp.	Caco-2, Hela	+		[341]
RVL (*Remusatia vivipara*)	breast cancer	+	+	[342]
ArtinM (*Artocarpus heterophyllus*)	Jurkat T cells	+	+	[70]
PCL (*Polygonatum cyrtonema*)	lung adenocarcinoma	+		[343]
	murine fibrosarcoma	+	+	[344]
	melanoma	+	+	[345]
	melanoma	+	+	[346]
AHA (*Arisema heterophyllum*)	lung cancer	+		[347]
LcA (*Lens culinaris*)	nasopharyngeal carcinoma	+		[348]
ASA (*Allium sativum*)	oral carcinoma	+		[349]
Con A (*Canavalia ensiformis*)	breast carcinoma	+		[350]
	leukemia	+	+	[351]
	glioblastoma	+	+	[352]
	ovarian cancer	+	+	[353]
	melanoma	+	+	[354]
SFL (*Sophora flavescens*)	HeLa cells	+		[59]
	breast carcinoma	+		[350]
CML (*Clematis montana*)	HeLa, breast cancer,	+		[83]
	hepatocellular carcinoma			
ConBr (*Canavalia brasiliensis*)	murine melanoma	+		[355]
	leukemia			[351]
PTA (*Pinellia ternata*)	hepatoma	+		[356]
ESA (*Eucheuma serra*)	osteosarcoma	+		[357]
	mice colon adenocarcinoma	+		[358]
	colon cancer, HeLa	+		[138]
OJL (*Ophiopogon japonicus*)	murine fibrosarcoma	+		[96]
LNL (*Liparis noversa*)	murine fribrosarcoma	+		[96]

## Abbreviations

ABA	*Agaricus bisporus* agglutinin
ACA	*Allium cepa* agglutinin
AMA	*Arum maculatum* agglutinin
ASA	*Allium sativum* agglutinin
Asp, D	Aspartic acid
Ath	*Arabidopsis thaliana*
ATP	Adenosine triphosphate
BMA	*Bowringia mildbraedii* agglutinin
CBA	Carbohydrate-binding agent
CBM	Carbohydrate-binding module
CBS	Carbohydrate-binding site
CEA	*Colocasia esculenta* agglutinin
Con A	Concanavalin A
CTL	*Centrolobium tomentosum* lectin
CVN	Cyanovirin N
EUL	*Euonymus europaeus* lectin
Glu, E	Glutamic acid
Gly, G	Glycine
GNA	*Galanthus nivalis* agglutinin
Heltuba	*Helianthus tuberosus* agglutinin
HHA	*Hippeastrum* hybdrid agglutinin
HIV	Human Immunodeficiency virus
HSP	Heat shock protein
Leu, L	Leucine
LcA	*Lens culinaris* agglutinin
LoL	*Lathyrus ochrus* lectin
MPA	*Maclura pomifera* agglutinin
MVL	*Microcystis viridis* lectin
OAA	*Oscillatoria agardhii* agglutinin
PAL	*Pterocarpus angolensis* lectin
PDB	Protein data bank
PHA	Phytohemagglutinin
Phe, F	Phenylalanine
PNA	Peanut agglutinin
PsA	*Pisum sativum* agglutinin
ROS	Reactive oxygen species
RIP	Ribosome inactivating protein
SBA	Soybean agglutinin
SNA	*Sambucus nigra* agglutinin
Trp, W	Tryptophane
Tyr, Y	Tyrosine
Val, V	Valine
VfA	*Vicia faba* agglutinin
